# HULC-IGF2BP2 Interaction Drives Proliferation and Metastasis in Colorectal Cancer

**DOI:** 10.7150/jca.101989

**Published:** 2024-10-28

**Authors:** Qiang Pang, Shansong Huang, Huiying Wang, Jiaqing Cao

**Affiliations:** 1 Department of Gastrointestinal Surgery, The Second Affiliated Hospital, Jiangxi Medical College, Nanchang University, Nanchang (330006), China; 2 Department of Rheumatology, Huzhou Central Hospital, Affiliated Central Hospital Huzhou University, Huzhou, China.

**Keywords:** Long non-coding RNA, HULC, IGF2BP2, Colorectal cancer

## Abstract

**Background:** Highly Upregulated in Liver Cancer (HULC) has been shown to play a pro-carcinogenic role in various cancer types. However, the specific molecular mechanisms of HULC in colorectal cancer (CRC) remain unclear. This study aimed to investigate the interaction between HULC and insulin-like growth factor 2 mRNA-binding protein 2 (IGF2BP2) in CRC and its role in promoting cancer progression.

**Methods:** We first assessed HULC expression levels in CRC patient tissues and adjacent normal tissues using RNA sequencing and fluorescence *in situ* hybridization (FISH). Functional assays, including cell proliferation, migration, and colony formation, were performed in CRC cell lines. To further explore the interaction between HULC and IGF2BP2, we conducted RNA immunoprecipitation (RIP), RNA pull-down, and immunofluorescence co-localization assays. Additionally, the role of HULC in CRC growth and metastasis was evaluated *in vivo* using a nude mouse xenograft model and a liver metastasis model.

**Results:** Our results showed that HULC was significantly overexpressed in CRC tissues and cell lines, with high HULC expression correlating with poor patient prognosis. HULC knockdown significantly inhibited CRC cell proliferation, migration, and epithelial-mesenchymal transition (EMT), while its overexpression enhanced these processes. RNA pull-down and RIP assays confirmed a direct interaction between HULC and IGF2BP2. Further experiments demonstrated that IGF2BP2 could reverse the inhibitory effects of HULC knockdown on CRC cell proliferation and migration, restoring the expression of CDK4, N-cadherin, and Vimentin. *In vivo* experiments revealed that HULC knockdown inhibited tumor growth and liver metastasis in nude mice, while HULC overexpression promoted malignant progression.

**Conclusion:** HULC promotes CRC cell proliferation, migration, and EMT through its interaction with IGF2BP2, highlighting the critical role of the HULC-IGF2BP2 axis in CRC progression. These findings identify HULC as a potential molecular target for CRC treatment and offer new insights for therapeutic strategies targeting CRC patients.

## Introduction

Colorectal cancer (CRC) is a malignant tumor that originates in the colon or rectum and is the most common malignancy of the digestive system. Research has shown that globally, CRC ranks as the third most prevalent type of cancer, accounting for approximately 10% of all cancer diagnoses, and is the second highest cause of cancer-related deaths, responsible for around 9.4% of all cancer fatalities^1^. Despite advancements in treatments like surgery, chemotherapy, and radiotherapy, the survival rate of CRC patients has increased. However, the main challenges in treatment and prognosis remain the proliferation of tumor cells and the development of metastasis^2^. Therefore, in-depth studies exploring the specific mechanisms of proliferation and metastasis in CRC are crucial for improving patient survival.

HULC (Highly Upregulated in Liver Cancer) is a novel lncRNA that was first found to be highly expressed in hepatocellular carcinoma tissues^3^, ^4^. Previously, Wang *et al.* found that HULC could promote liver cancer progression through miR675-PKM2 pathway^3^. Zhou *et al.* showed that HULC could promote breast cancer metastasis by targeting IGF1R and affecting the PI3K-AKT axis^5^. Studies have also shown that HULC can enhance the advancement and spread of gastric cancer by suppressing miR-488^6^. Additionally, research has shown that HULC may enhance the advancement and spread of gastric cancer by suppressing miR-488. While the involvement of HULC in various cancer types like hepatocellular carcinoma, gastric cancer, and osteosarcoma is well-established, its function in CRC remains unclear. Therefore, further research is needed to fully understand the specific role and mechanism of HULC in the development of CRC.

Recent discoveries have shown that lncRNAs are capable of interacting with RNA-binding proteins (RBPs), leading to their involvement in various cellular processes^7^-^9^. IGF2BP2, a new RNA-binding protein, is highly expressed in various cancers and has been found to support tumor growth and progression^10^-^12^. Wang *et al.* found that IGF2BP2 interacted with the LncRNA LINRIS and promoted aerobic glycolysis in CRC^13^. However, whether HULC promotes CRC progression by interacting with IGF2BP2 is unknown.

The study initially discovered through bioinformatics analysis that HULC was significantly upregulated in colorectal cancer patients with metastasis. Subsequent *in vivo* and *in vitro* studies validated that altering the levels of HULC could suppress or enhance the growth and spread of colorectal cancer. Additionally, it was discovered that HULC has the ability to enhance the growth and spread of CRC through its interaction with IGF2BP2. The discovery indicates that HULC plays a role in the progression of CRC and could be a promising focus for CRC therapy.

## Material and methods

### Tissue samples

The Ethics Committee of the Second Affiliated Hospital of Nanchang University approved all experiments. In 2023, samples from 80 patients with CRC were collected at the Second Affiliated Hospital of Nanchang University, including both cancerous and normal tissues from each patient. The inclusion criteria were: (1) patients with a first-time diagnosis of colorectal cancer who had not received any prior treatment; (2) no history of other malignancies; and (3) no severe infections, liver disease, or other systemic diseases. The exclusion criteria were: (1) presence of other severe diseases such as diabetes or cardiovascular disease; and (2) patients who had previously undergone radiotherapy or chemotherapy. Samples were stored at -80°C or in paraformaldehyde fixation for preservation. All participants gave their informed written consent.

### Cell culture and cell transfection

NCM460, SW480, HT29, SW620, DLD1 and HCT116 cell lines were purchased from Fu Heng Biologicals. NCM460, SW480, HT29, SW620 and DLD1 were cultured in DMEM medium, while HCT116 was cultured in RMPI-1640 medium. Each medium was supplemented with 10% fetal bovine serum and 1% dual antibody, then placed in an incubator at 37°C with 5% CO_2_.

Lentiviruses for HULC knockdown (shHULC) and overexpression (HULC) and IGF2BP2 overexpression (IGF2BP2) and their negative controls (shNC and Vector) were obtained from General Biologicals, and small interfering fragments targeting IGF2BP2 (siIGF2BP2) were obtained from General Biologicals. Transfection of lentivirus and interference fragments was performed according to the instructions for lentivirus and interference fragments.

### RNA extraction and qPCR

Total RNA was extracted from tissue samples and cells using TRIzol reagent (Invitrogen, USA). The cDNA was then created with the RevertAid First Strand cDNA Synthesis Kit from Thermo in the United States. The quantification of cDNA was performed with SYBR Green qPCR Master Mix (Servicebio, China) in real-time PCR to assess the levels of HULC expression. The subsequent initiators employed were HULC 5′-AACAGACCAAAGCATCAAGCA-3′ (forward) and 5′-TTGCCACAGGTTGAACACTTA-3′ (reverse); GAPDH 5′-GGAAGCTTGTCATCAATGGAAATC-3′ (forward) and 5′-TGATGACCCTTTTGGCTCCC-3′ (reverse).

### Proliferation experiment

For Edu assay: the treated cells were inoculated in 96-well plates at 20,000 per well, and after 24 hours, the cells were operated according to the instructions of EdU assay, photographed and analyzed by fluorescence microscope. The plate cloning experiment involved inoculating cells into six-well plates at a density of 800 cells per well, incubating them for approximately 14 days, and subsequently staining and quantifying colony formation with crystal violet.

### Migration assay

In the Transwell experiment, the CRC cells treated with serum-free medium were suspended and placed in the upper chamber, while 600 μl of medium with 10% FBS was added to the lower chamber. The cells were then incubated for 48 hours. Next, crystal violet was used to stain the cells before counting them. In experiments to study wound healing, CRC cells were evenly distributed on 6-well plates and allowed to grow until fully confluent. The cell monolayer was then gently scratched using a 200ul sterile pipette tip. At 0 and 48 hours after scratching the cells, cell migration at the same location was examined under a microscope and the scratched area was assessed using ImageJ.

### Western blotting

Proteins from cells were isolated by utilizing RIPA lysis buffer with protease and phosphatase inhibitors. The BCA protein assay kit was used to measure the protein concentration. Proteins were separated using a 10% SDS-PAGE gel and then transferred onto a PVDF membrane. Following a 2-hour incubation period with 5% skim milk, the membranes were then exposed to primary antibodies overnight at 4°C. Proteintech (Wuhan, China) provided the primary antibodies GAPDH, IGF2BP2, E-cadherin, N-cadherin, Vimentin, CDK4, and CDK6.

### Fluorescence *in situ* hybridization (FISH)

The HULC-specific fluorescent probe was purchased from Servicebio. The tissues and cells were treated with 4 % paraformaldehyde, followed by permeabilization using 0.5% Triton X-100, and then exposed to the anti-HULC probe. Cell nuclei were stained using DAPI. After rinsing again, slides were fixed using Diamond Antifade sealer. Finally, they were placed under an orthogonal fluorescence microscope for observation and image acquisition.

### Immunofluorescence (IF)

To perform cellular immunofluorescence, cells were initially treated with 4% paraformaldehyde. Next, the serum was contained at ambient temperature. Cells were treated with antibodies and left to incubate overnight at 4°C. Next, and secondary antibody with fluorescent labeling was added to the cells. Finally, the cells were stained with DAPI cytosolic solution and observed and images were captured using an orthogonal fluorescence microscope.

### RNA pull-down assay

The HULC full-length vector was created, with the full-length sequence of the HULC plasmid serving as a guide for PCR amplification to produce the T7 promoter sequence on both DNA strands, which was then utilized for generating the RNA pull down probe through *in vitro* transcription. First, biotinylated RNA was transcribed *in vitro*. Subsequently, streptavidin affinity magnetic beads were employed to bind to the biotin-labeled RNA. Finally, proteins were eluted and analyzed by mass spectrometric silver staining and western blotting.

### RNA Immunoprecipitation (RIP)

The specific procedure of the RIP experiment was carried out with reference to the Giese Biological RIP kit. First, the magnetic beads are eluted and conjugated with the appropriate antibodies, followed by adequate lysis of colorectal cancer cells to obtain lysates. RNA is purified by incubating the lysate with the magnetic bead-antibody complex overnight at a temperature of 4°C. Finally, expression is verified using qPCR.

### Animal studies

Male nude mice of the BALB/c strain, aged 3-4 weeks, were acquired from Beijing Specific Biological Biotechnology Co. Approval for all animal-related research was obtained from the Animal Ethics Committee of Nanchang University. All experiments involving animals followed the guidelines outlined in the Manual for the Treatment and Handling of Laboratory Animals.

To establish the subcutaneous tumor model, colorectal cancer cell lines with stable knockdown or overexpression were created and then injected into nude mice at a concentration of 5-10 million cells per 200ul.There were 4 groups, each containing 5 nude mice. The nude mice were left to be executed by CO_2_ after 3-4 weeks, and then the subcutaneous tumors of the nude mice were excised, the tumor volume was measured, and the tumors were weighed.

In the CRC liver metastasis model, the cells were initially suspended in PBS to modify the cell concentration to 1 million cells per 50 μL. Subsequently, the nude mice were anesthetized, shaved and disinfected, and the spleens were exposed, and the needles were inserted along the spleens at a distance of about 1.5 cm, and 50 μL of cell suspension was injected slowly, and then the wounds were closed by suturing. After 1 month of normal feeding, the nude mice were executed by CO_2_ and the liver tissues were removed and photographed for analysis.

### Statistical analysis

Data are shown as mean ± standard deviation (SD). The two groups were compared using either two-tailed unpaired or paired Student's t-test. Comparisons between multiple groups were analyzed by one-way ANOVA. Data were statistically analyzed using GraphPad 9.0.2 software. Statistically significant results were defined as having a *P* value below 0.05.

## Results

### HULC is highly expressed in CRC

**Figure 1:**
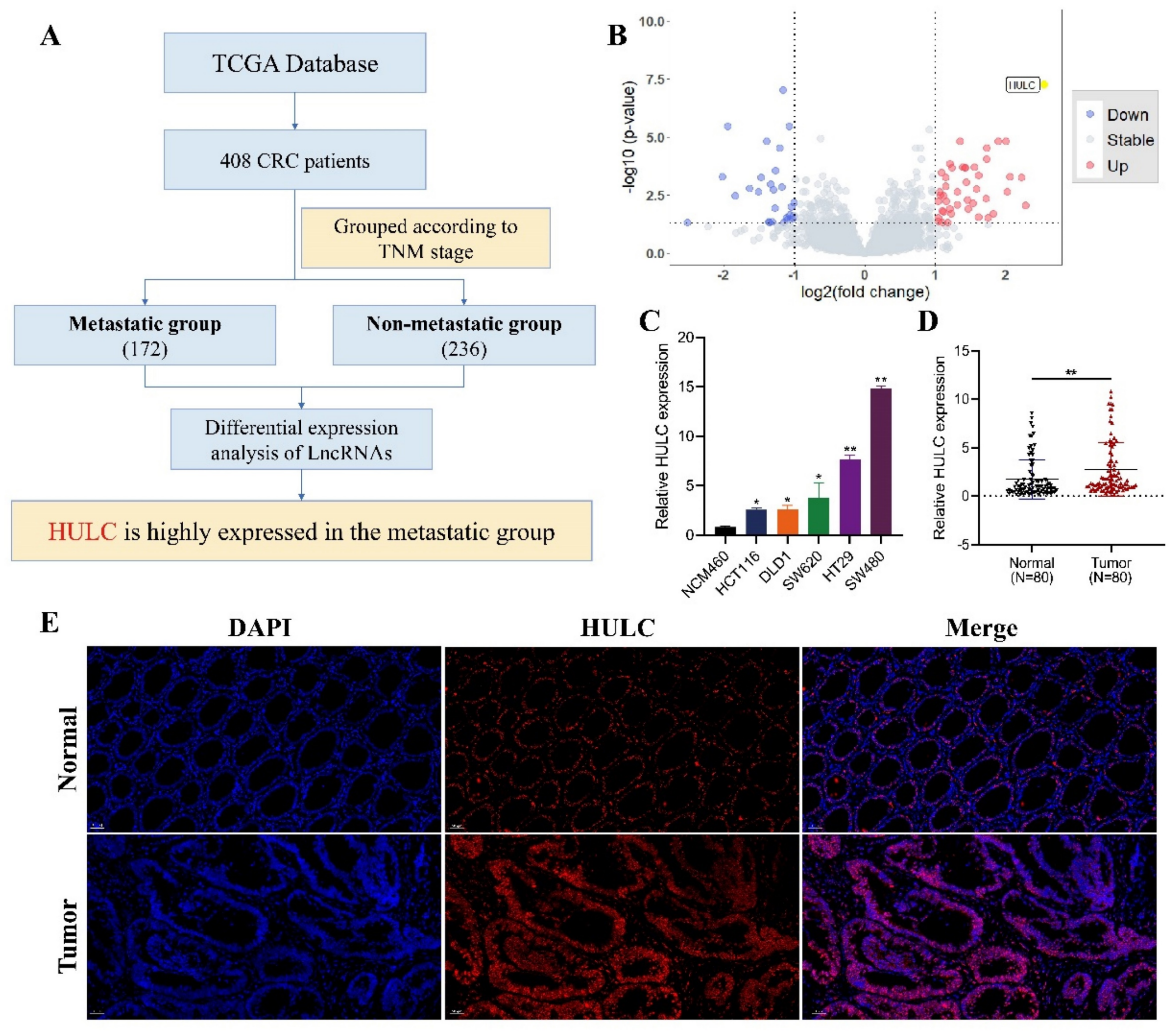
HULC is highly expressed in colorectal cancer (CRC). A, Flowchart of the source of HULC.B, Bioinformatics identification of HULC and presented by volcano plot. C, qPCR detection of HULC expression in CRC cell lines. D, qPCR detection of HULC expression in CRC tissues. E, FISH detection of HULC expression in CRC tissues. *P < 0.05, **P < 0.01. Image scale is: 50 uM.

Flowchart of HULC as the main molecular source of this study (Fig. 1A). The volcano plot demonstrates that HULC is highly expressed in metastatic group of CRC patients (Fig. 1B). HULC up-regulation was confirmed in CRC cell lines, and its expression was further validated through qPCR on tumor tissues and adjacent normal tissues from 80 CRC patients, revealing elevated levels of HULC in CRC (Fig. 1C-D). Furthermore, FISH analysis was conducted with a HULC fluorescent probe on tumor tissues and adjacent normal tissues from patients with CRC, revealing high expression of HULC in CRC (Fig. 1E). Finally, we stratified 80 CRC patients into high and low HULC expression groups and analyzed the correlation between HULC expression and clinical characteristics. High HULC expression was positively associated with larger tumor size, distant metastasis, and higher tumor grade (Table 1).

### HULC promotes the proliferative capacity of CRC cells

**Figure 2:**
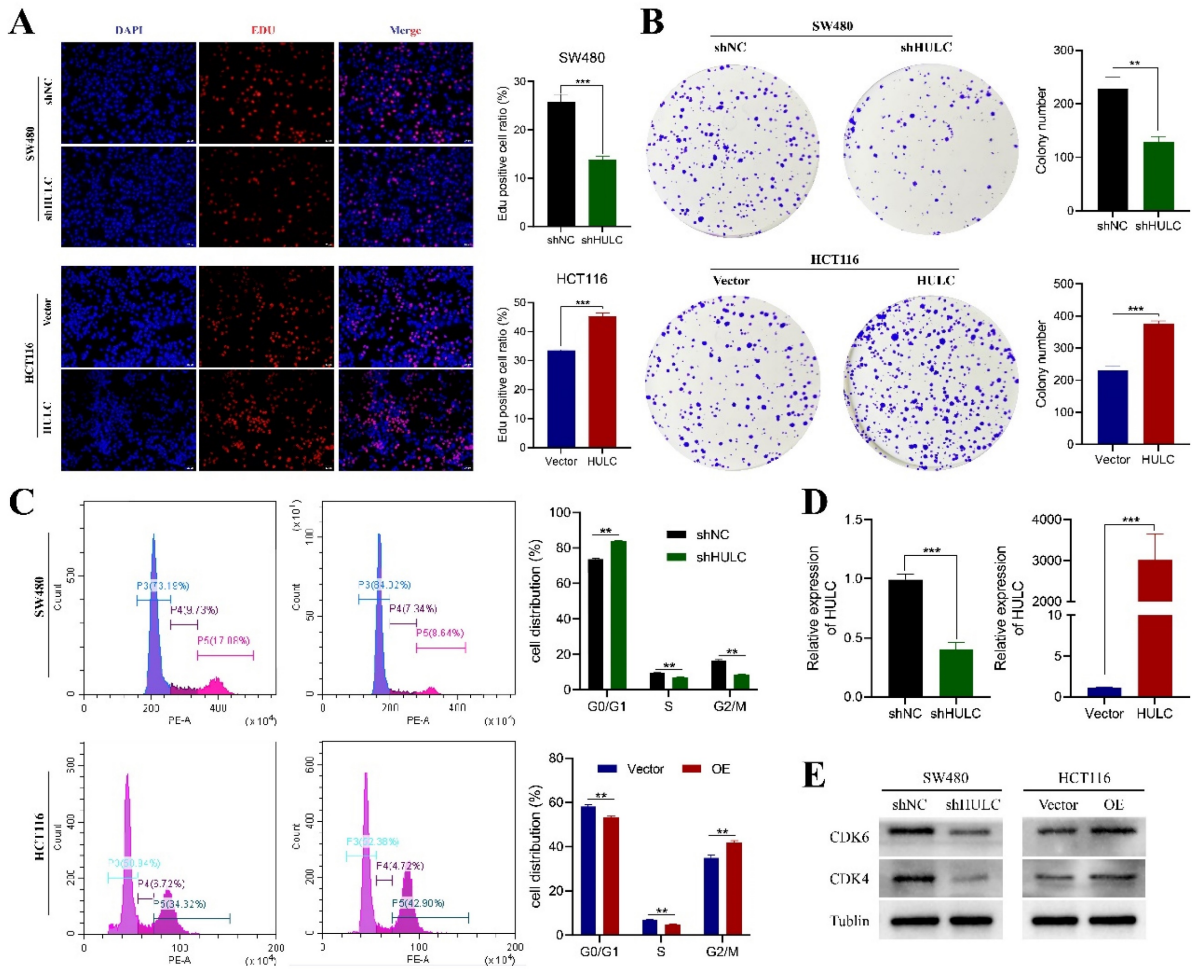
HULC promotes the proliferation of CRC cells. A-B, Edu assay and plate cloning assay to assess the effect of HULC knockdown or overexpression on the proliferative capacity of CRC cells. C, Flow cycle cytometry to detect the effect of shHULC or HULC on the cell cycle. D, qPCR to assess the efficiency of HULC knockdown or overexpression. E, western blotting to assess the effect of shHUL or HULC on the cycle protein expression. *P < 0.05, **P < 0.01, ***P < 0.001. Image scale is: 20 uM.

Considering that HULC expression is highest in SW480 cell line and relatively low in HCT116. We established the SW480 cell line with stable knockdown of HULC by transfection of shNC and shHULC, and constructed the HCT116 cell line with stable overexpression of HULC by transfection of Vector and HULC for experimental follow-up studies. qPCR analysis revealed a decrease in HULC expression in stably transfected cell lines following shHULC transfection, as compared to the shNC group. In contrast, the levels of HULC expression significantly increased following HULC overexpression in HCT116 cells, as shown in Fig. 2D-E. The Edu analysis indicated that reducing HULC expression greatly reduced the growth of SW480 cells, whereas increasing HULC expression significantly boosted the growth of HCT116 cells (Fig. 2A). In addition, the number of cells in the plate clone formation assay of SW480 cells was significantly reduced after knocking down HULC, while the number of clones of HCT116 cells was significantly increased when HULC was overexpressed (Fig. 2B).

To further analyze the role of HULC in the proliferative viability of CRC cells, we performed flow cytometry. The results of the cell cycle assay showed that SW480 after knockdown of HULC mainly stagnated in the G0/G1 phase, whereas HCT116 cells transformed more toward the G2/M phase when overexpressing HULC (Fig. 2C). Furthermore, the levels of CDK4 and CDK6 proteins were reduced in SW480 cells following HULC knockdown, while HULC upregulation led to an increase in CDK4 and CDK6 protein expression in HCT116 cells (Fig. 2E).

**Figure 3:**
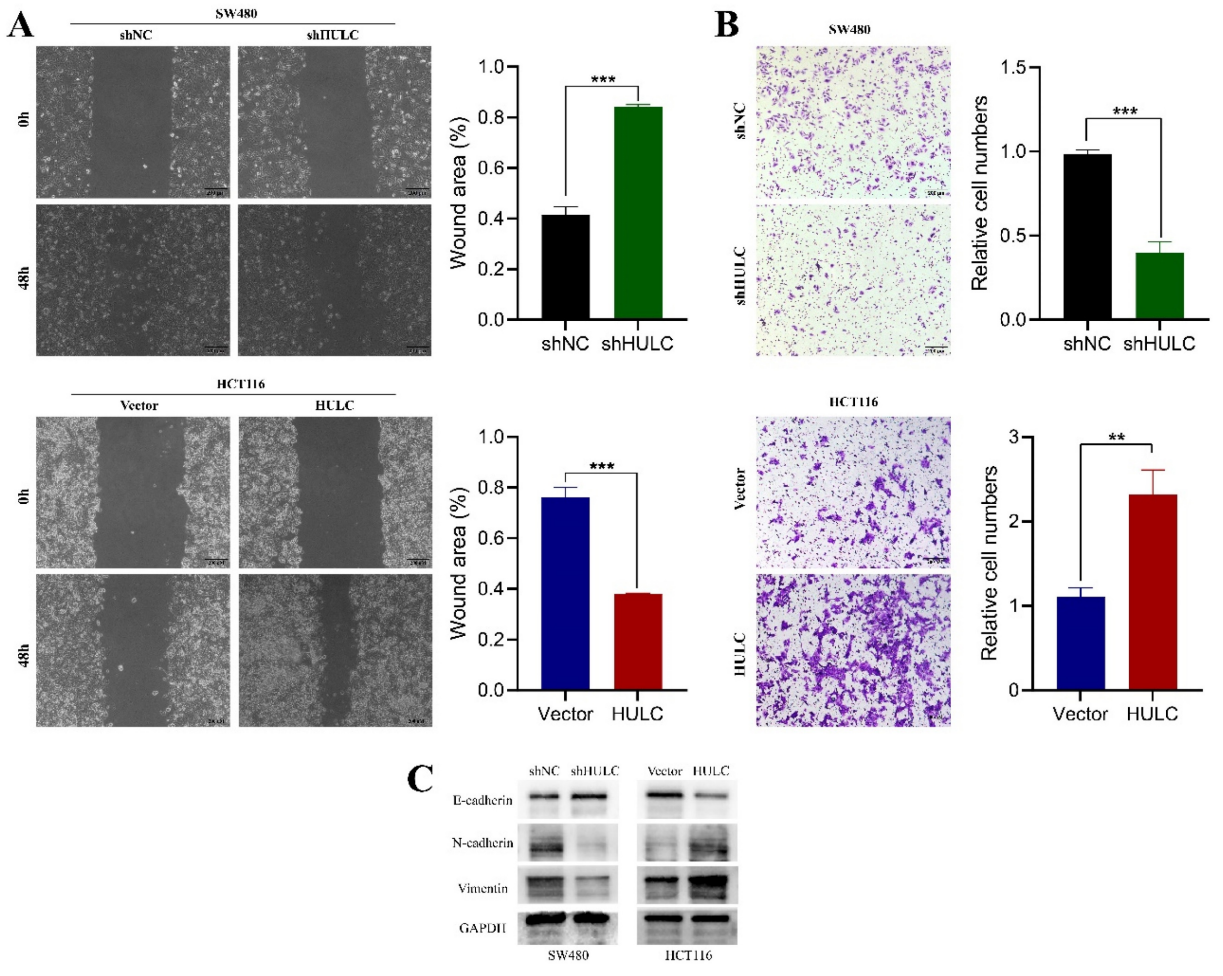
HULC promotes CRC cell migration and EMT. A. Wound healing assay was performed to assess the migratory ability of shHULC or HULC on CRC cells. B. Transwell assay was performed to detect the migratory ability of CRC cells. C. Western blotting was performed to assess the expression of EMT-associated molecules by shHULC or HULC. *P < 0.05, **P < 0.01, ***P < 0.001. Image scale is: 200 uM.

HULC enhances the movement and transition of CRC cells from epithelial to mesenchymal to further explore the role of HULC in CRC cells, we performed wound healing assay and Transwell assay. The findings indicated a notable decrease in the migration rate of SW480 cells 48 hours after HULC knockdown, whereas the migration rate of HCT116 cells significantly rose upon HULC overexpression (Fig. 3A). Transwell assay results showed a decrease in migrated SW480 cells after HULC knockdown, and an increase in migrated HCT116 cells with HULC overexpression (Fig. 3B). After analyzing the molecular proteins linked to EMT, we discovered a notable rise in E-cadherin expression and a decrease in N-cadherin and vimentin expression in SW480 cells following HULC knockdown. Conversely, HULC overexpression led to a decrease in E-cadherin expression and an increase in N-cadherin and vimentin expression (Fig. 3C). Collectively, these findings suggest that HULC can enhance the growth and movement of CRC cells and the transition of CRC cells to a mesenchymal state.

### HULC plays a role in promoting CRC proliferation and metastasis *in vivo*

**Figure 4:**
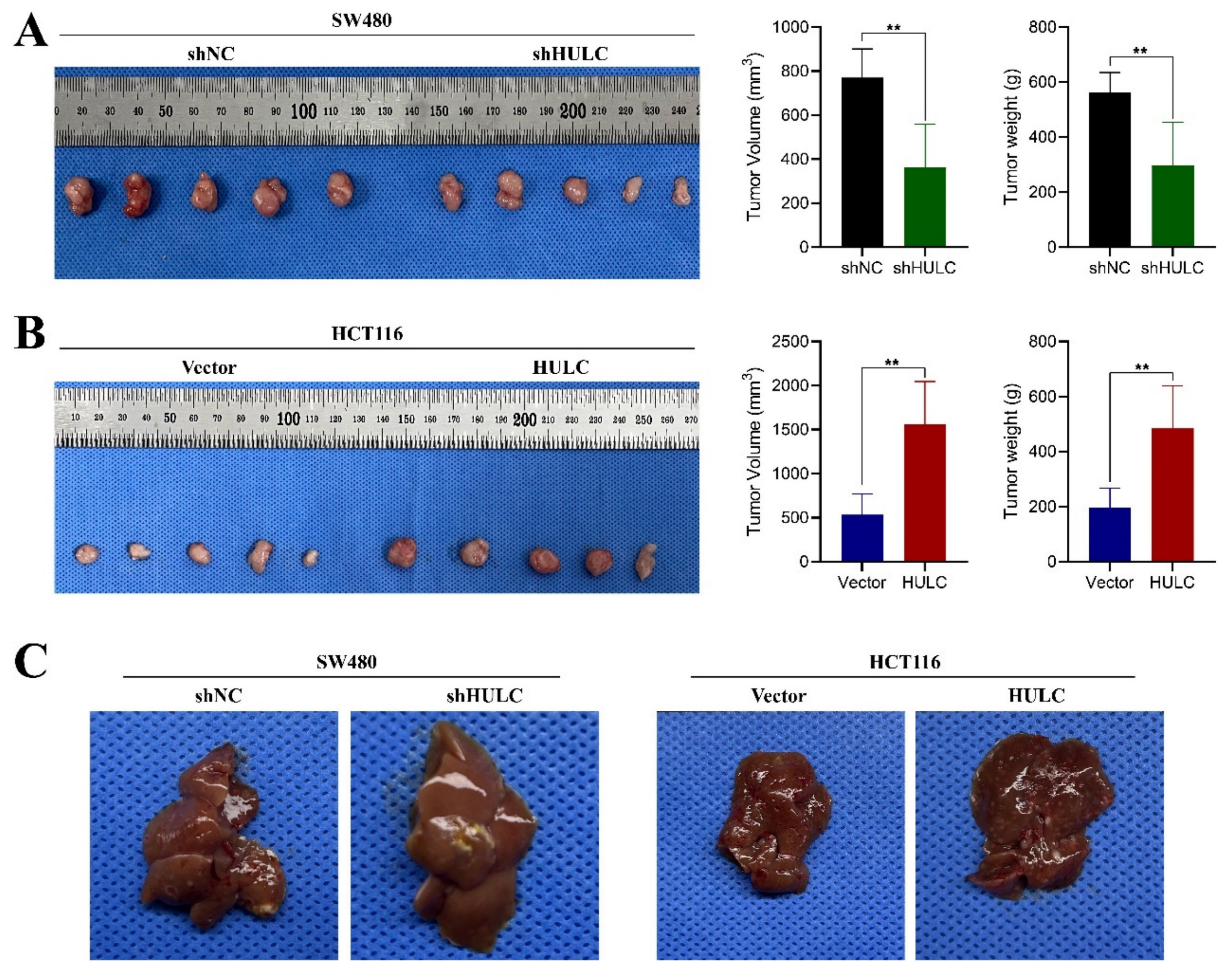
HULC promotes CRC growth and metastasis *in vivo*. A, Effect of knockdown of HULC on the growth of CRC cells *in vivo*. B, Effect of overexpression of HULC on the growth of CRC cells *in vivo*. C, Effect of shHULC or HULC on the liver metastasis of CRC cells *in vivo*. *P < 0.05, **P < 0.01, ***P < 0.001.

In order to assess the biological function of HULC in colorectal cancer in living organisms, we developed a subcutaneous xenograft model using nude mice. The mice were injected with SW480 cell lines that were stabilized with shNC and shHULC, as well as Vector and HULC. Downregulation of HULC led to a notable decrease in both tumor volume and weight, as well as a reduced tumor growth rate when compared to the shNC group (Fig. 4A). Interestingly, the above results were completely opposite after HULC overexpression (Fig. 4B). The findings indicate that HULC functions as a cancer-causing LncRNA that enhances cell growth and aids in the advancement of CRC in living organisms.

In addition, we established a liver metastasis model of CRC cells. Metastatic lesions in the livers of mice showed a notable decrease following the suppression of HULC, while there was a significant rise in metastatic lesions in the livers following the upregulation of HULC.

### HULC can directly interact with IGF2BP2

**Figure 5:**
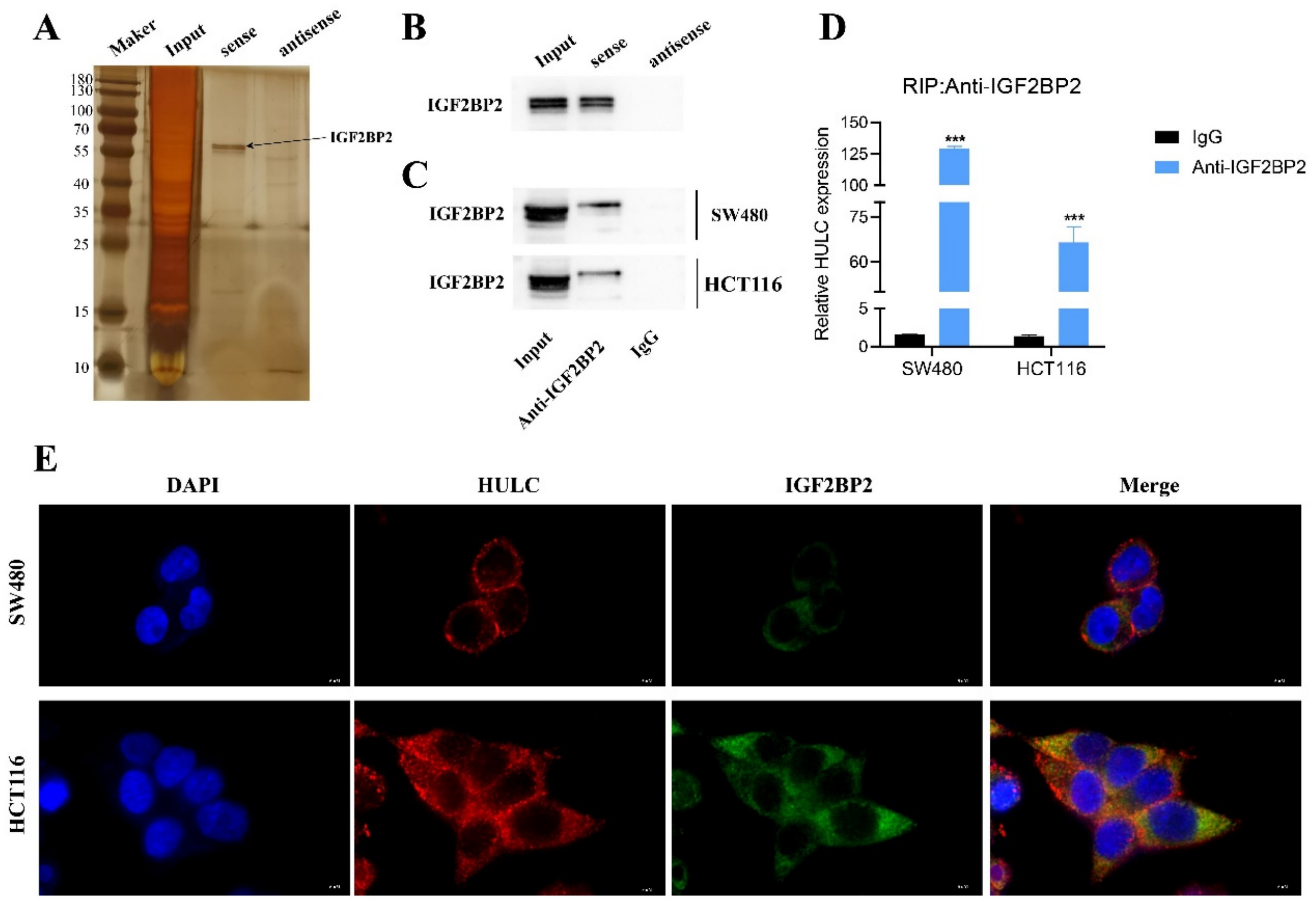
HULC binding to IGF2BP2. A, RNA pull down assay for silver staining to identify IGF2BP2. B, RNA pull down assay to detect the interaction of IGF2BP2 with HULC. C-D, RNA immunoprecipitation assay (RIP) to assess the binding relationship between IGF2BP2 and HULC. e. Immunofluorescence co-localization to assess the HULC binding to IGF2BP2. ***P < 0.001. Image scale is: 5 uM.

In order to clarify the potential role of HULC in CRC, we created a complete biotin-labeled HULC sense and antisense sequence probe, conducted RNA pull down and silver staining experiments, and identified distinct bands (Fig. 5A). Subsequently, the HULC justice chain was found to be enriched for IGF2BP2 protein rather than the antisense chain by western blotting (Fig. 5B). Subsequently, it was also confirmed that IGF2BP2 could enrich HULC by RIP assay (Fig. 5C-D). Furthermore, the immunofluorescence co-localization analysis revealed that HULC and IGF2BP2 were co-localized in the cytoplasm (Fig. 5E). These results suggest that HULC can interact with IGF2BP2.

### IGF2BP2 rescues the biological behavior of HULC

**Figure 6:**
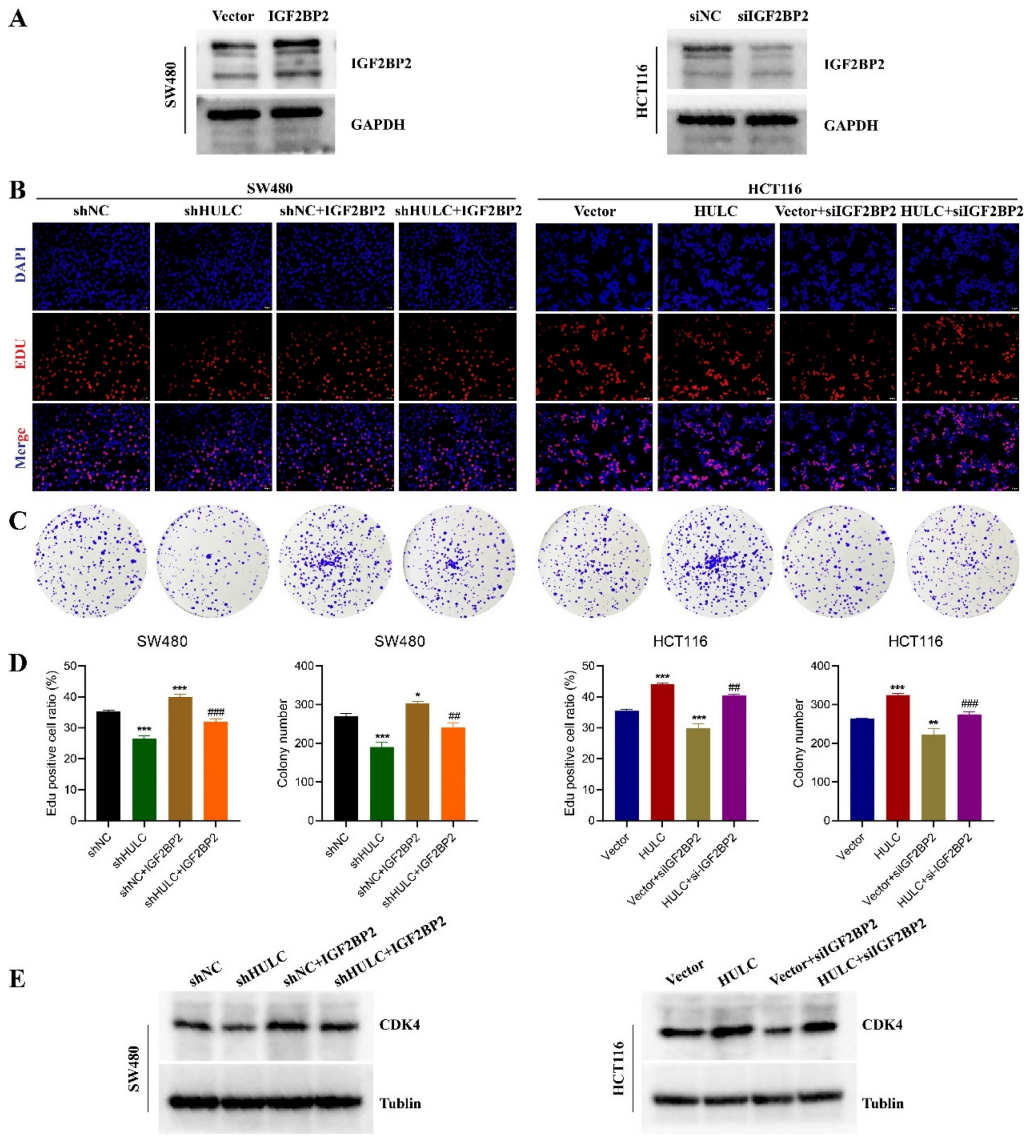
HULC interacts with IGF2BP2 to promote CRC cell proliferation. Overexpression of IGF2BP2 on the basis of shHULC; knockdown of IGF2BP2 on the basis of HULC. A, western blot to detect the efficiency of knockdown or overexpression of IGF2BP2. B-D, Edu assay and plate cloning assay to detect the cell proliferative ability. E, western blot to detect the CDK4 expression. *P < 0.05, **P < 0.01, ***P < 0.001. Image scale is: 20 uM.

To explore the role of IGF2BP2 in HULC promoting CRC progression. Initially, we confirmed the effectiveness of reducing and increasing IGF2BP2 levels, with the outcomes indicating that the interference segment notably decreased IGF2BP2 expression, whereas lentiviral transfection notably elevated IGF2BP2 levels (Fig. 5A). Subsequently, we transfected IGF2BP2 overexpressing lentivirus together with shNC or shHULC in SW480 cells. It was found that knockdown of HULC inhibited the proliferation of SW480 cells, while IGF2BP2 overexpression reversed this effect (Fig. 6B-D). In addition, knockdown of HULC decreased CDK4 protein expression in SW480 cells, while IGF2BP2 overexpression also reversed this expression pattern (Fig. 6E). Similarly, siIGF2BP2 was transfected with Vector or HULC in HCT116 cells, and it was found that knockdown of IGF2BP2 could reverse the cell proliferation-promoting effect of HULC, and CDK4 protein expression could also be reversed by knockdown of IGF2BP2 (Fig. 6B-E).

**Figure 7:**
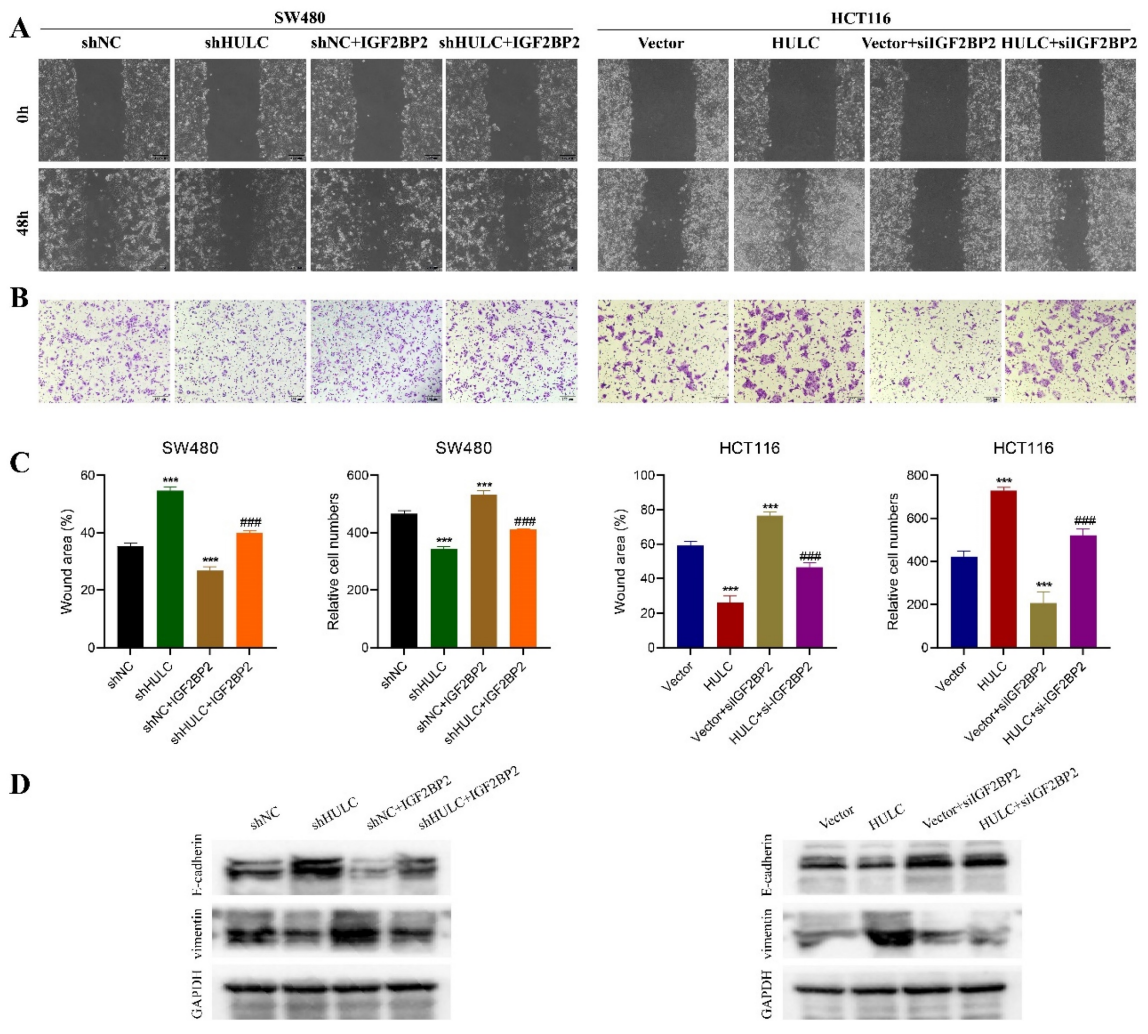
HULC interacts with IGF2BP2 to promote CRC cell migration and EMT. A-C, Wound healing assay and Transwell assay were performed to detect the migratory ability of CRC cells. D, western blot was performed to detect the expression of E-cadherin and Vimentin. *P < 0.05, **P < 0.01, ***P < 0.001. Image scale is: 200 uM.

IGF2BP2 overexpression reversed the inhibitory effect of HULC knockdown on SW480 cell migration, as shown in the Fig. 7A-C. Silencing of HULC led to an upregulation of E-cadherin protein in SW480 cells and a downregulation of Vimentin, which was then reversed by the overexpression of IGF2BP2 (Fig. 7D). Similarly, knockdown of IGF2BP2 was found to reverse the cell migration-promoting effect of HULC in HCT116 cells, and the expression pattern of E-cadherin and Vimentin proteins was also reversed by knockdown of IGF2BP2 (Fig. 7A-D).

## Discussion

From the human genome as a whole, only 2% of genes have coding proteins, while most of them belong to non-coding RNAs^14^. LncRNAs, being the primary elements of non-coding RNAs, have a crucial impact on the formation and progression of tumors^15^-^17^. Our study revealed that HULC exhibited high levels of expression in CRC tissues as well as in cell lines. Manipulating HULC levels *in vitro* had a substantial impact on the growth, movement, and transformation of CRC cells, as well as on tumor development and metastasis in animal models. In addition, our study also found that HULC could bind to IGF2BP2 and thus regulate the proliferation, migration and EMT of CRC cells.

Prior research has indicated that HULC is markedly increased and serves a crucial oncogenic function in various types of cancers, such as liver cancer^18^, stomach cancer^19^ and CRC^20^. Although HULC has been reported to be highly expressed in CRC and shown to promote the malignant progression of CRC, most previous studies have been limited to tissue sample and cell line validation^21^-^24^. The specific molecular mechanisms through which HULC drives CRC proliferation, EMT transformation, and liver metastasis remain largely unexplored. In this study, we confirmed the oncogenic role of HULC in CRC by *in vivo* experiments. To further explore the role of HULC in promoting CRC progression, we analyzed RNA pull down and RIP experiments and identified that IGF2BP2 can bind to HULC. Several research studies have demonstrated the ability of LncRNAs to interact with RNA-binding proteins, leading to significant contributions in the oncology field^7^, ^17^, ^25^. IGF2BP2, functioning as an RNA-binding protein, plays a part in controlling the positioning, durability, and interpretation of RNA molecules^26^, ^27^. Previous studies have indicated that the LncRNA AGAP2-AS1 plays a role in advancing bladder cancer by interacting with IGF2BP2^28^. Wang *et al.* study found that LINRIS binds to IGF2BP2 to regulate the MYC axis to promote CRC progression^13^. In our study, both HULC and IGF2BP2 were found to be predominantly distributed in the cytoplasm by immunofluorescence co-localization analysis, corroborating that HULC and IGF2BP2 bind to each other to play a role. Rescue experiments also confirmed that HULC interacts with IGF2BP2 to further promote CRC progression.

Nevertheless, there are still some constraints in our work. For example, the specific molecular mechanism between HULC and IGF2BP2 is not clear; considering that HULC expression does not affect IGF2BP2 changes, we hypothesize that HULC may play a pro-cancer role by recruiting IGF2BP2 and thus, but this requires further studies to explore this mechanism. In addition, the effect of HULC interaction with IGF2BP2 on the downstream mechanisms also needs to be further investigated.

## Conclusion

Overall, our study demonstrates that HULC promotes CRC proliferation, EMT transformation, and liver metastasis through its interaction with the RNA-binding protein IGF2BP2. This finding identifies HULC as a potential molecular target for CRC treatment and provides deeper insights into the molecular mechanisms driving HULC-mediated malignant progression in CRC.

## Figures and Tables

**Table 1:** Relationship between HULC expression and clinicopathologic Characteristics in patients.

Characteristics	High expression of HULC	Low expression of HULC	*P* value
n	40	40	
Gender, n (%)			0.823
Female	22 (27.5%)	21 (26.2%)	
Male	18 (22.5%)	19 (23.8%)	
Age, n (%)			0.502
>65	22 (27.5%)	19 (23.8%)	
≤ 65	18 (22.5%)	21 (26.2%)	
Diameter of tumor(cm), n (%)			0.025*
≤ 5	14 (17.5%)	24 (30%)	
>5	26 (32.5%)	16 (20%)	
Lymphatic metastasis, n (%)			0.073
Negative	15 (18.8%)	23 (28.7%)	
Positive	25 (31.2%)	17 (21.2%)	
Distant metastasis, n (%)			0.043*
Negative	32 (40%)	38 (47.5%)	
Positive	8 (10%)	2 (2.5%)	
TNM stage, n (%)			0.012*
I/II	11 (13.8%)	22 (27.5%)	
III/IV	29 (36.2%)	18 (22.5%)	
